# Genomic Context of Azole Resistance Mutations in *Aspergillus fumigatus* Determined Using Whole-Genome Sequencing

**DOI:** 10.1128/mBio.00536-15

**Published:** 2015-06-02

**Authors:** Alireza Abdolrasouli, Johanna Rhodes, Mathew A. Beale, Ferry Hagen, Thomas R. Rogers, Anuradha Chowdhary, Jacques F. Meis, Darius Armstrong-James, Matthew C. Fisher

**Affiliations:** ^a^ National Heart & Lung Institute, Imperial College London, London, United Kingdom; ^b^ Department of Medical Microbiology, Charing Cross Hospital, Imperial College Healthcare NHS Trust, London, United Kingdom; ^c^ Department of Infectious Disease Epidemiology, Imperial College London, London, United Kingdom; ^d^ Institute of Infection and Immunity, St. George’s University of London, London, United Kingdom; ^e^ Department of Medical Microbiology and Infectious Diseases, Canisius Wilhelmina Hospital, Nijmegen, The Netherlands; ^f^ Department of Clinical Microbiology, Trinity College Dublin, Dublin, Ireland; ^g^ St. James’ Hospital, Dublin, Ireland; ^h^ Department of Medical Mycology, Vallabhbhai Patel Chest Institute, University of Delhi, Delhi, India; ^i^ Department of Medical Microbiology, Radboud University Medical Center, Nijmegen, The Netherlands; University of California

## Abstract

A rapid and global emergence of azole resistance has been observed in the pathogenic fungus *Aspergillus fumigatus* over the past decade. The dominant resistance mechanism appears to be of environmental origin and involves mutations in the *cyp51A* gene, which encodes a protein targeted by triazole antifungal drugs. Whole-genome sequencing (WGS) was performed for high-resolution single-nucleotide polymorphism (SNP) analysis of 24 *A. fumigatus* isolates, including azole-resistant and susceptible clinical and environmental strains obtained from India, the Netherlands, and the United Kingdom, in order to assess the utility of WGS for characterizing the alleles causing resistance. WGS analysis confirmed that TR_34_/L98H (a mutation comprising a tandem repeat [TR] of 34 bases in the promoter of the *cyp51A* gene and a leucine-to-histidine change at codon 98) is the sole mechanism of azole resistance among the isolates tested in this panel of isolates. We used population genomic analysis and showed that *A. fumigatus* was panmictic, with as much genetic diversity found within a country as is found between continents. A striking exception to this was shown in India, where isolates are highly related despite being isolated from both clinical and environmental sources across >1,000 km; this broad occurrence suggests a recent selective sweep of a highly fit genotype that is associated with the TR_34_/L98H allele. We found that these sequenced isolates are all recombining, showing that azole-resistant alleles are segregating into diverse genetic backgrounds. Our analysis delineates the fundamental population genetic parameters that are needed to enable the use of genome-wide association studies to identify the contribution of SNP diversity to the generation and spread of azole resistance in this medically important fungus.

## IMPORTANCE

Resistance to azoles in the ubiquitous ascomycete fungus *A. fumigatus* was first reported from clinical isolates collected in the United States during the late 1980s. Over the last decade, an increasing number of *A. fumigatus* isolates from the clinic and from nature have been found to show resistance to azoles, suggesting that resistance is emerging through selection by the widespread usage of agricultural azole antifungal compounds. Aspergillosis is an emerging clinical problem, with high rates of treatment failures necessitating the development of new techniques for surveillance and for determining the genome-wide basis of azole resistance in *A. fumigatus*.

## INTRODUCTION

Fungal infections are increasingly recognized as a threat to human (1, 2), animal, and plant (3) populations. *Aspergillus* species are ubiquitous, saprophytic fungi with airborne conidia that grow on organic matter. *Aspergillus fumigatus* is the principal causative agent of human aspergillosis, which can range from allergic syndromes to invasive aspergillosis (IA), a life-threatening infection in immunocompromised hosts. Profoundly neutropenic patients receiving chemotherapy or hematopoietic stem cell or solid organ transplantation are at high risk of IA (4). Oral triazole antifungal drugs (itraconazole, voriconazole, and posaconazole) are effective against *A. fumigatus* and remain the front-line therapy in the management and prophylaxis of IA (5). However, the emergence of azole resistance in *A. fumigatus* isolates from Europe, Asia, the Middle East, and recently, Africa (6–8) is a global and evolving public health problem (9), although surprisingly, the United States seems to be exempt from this increase in azole resistance (10). Treatment failure due to azole-resistant strains is a major clinical concern in the management of patients with IA, with high rates of patient mortality being observed (11–13).

The true prevalence of azole resistance in *Aspergillus* species is largely unknown; however, frequencies of resistance varying between 0 and 6% have been determined for different clinical centers (14). Azole-resistant *A. fumigatus* strains have been isolated from both azole-naive and -exposed patients, as well as broadly from the natural environment in several countries. This has led to the hypothesis that the widespread use of structurally similar azoles in agriculture has led to increases in resistance in the clinical context (9, 15, 16).

The molecular basis of resistance to triazoles in *A. fumigatus* mainly involves point mutations at several codons in the *cyp51A* gene, which encodes lanosterol 14-α-sterol demethylase, combined with tandem repeats in the promoter region (17, 18). This enzyme is required for the biosynthesis of ergosterol, an essential component of the fungal cell membrane. Single-nucleotide polymorphisms (SNPs) in *cyp51A* cause structural alterations due to amino acid substitutions. Numerous SNPs that confer increased resistance to triazoles *in vitro* have been reported in this gene in *A. fumigatus* (19–23). However, a predominant resistance mutation, TR_34_/L98H, consists of a tandem repeat (TR) of 34 bases in the promoter of the *cyp51A* gene and a leucine-to-histidine change at codon 98 (24); this mutation is globally widespread in environmental isolates of the fungus. Recently, a novel *cyp51A*-mediated resistance mutation that leads to high-level voriconazole resistance, TR_46_/Y121F/T289A, has been described as occurring in environmental and clinical isolates of *A. fumigatus* found in the Netherlands (25), Belgium (26), Denmark (27), Germany (28), Tanzania (7), and India (29).

Typing methods, such as multilocus sequence typing (MLST), have fallen out of favor due to their low discriminatory power and considerable cost (30); the *Aspergillus fumigatus* MLST database is no longer curated, and higher-resolution methods are becoming more widely accessible for the analysis of small eukaryotic genomes due to falling costs (31). Driven by rapid technological advances, the application of whole-genome sequencing (WGS) to type microbial genomes is increasingly used to improve patient care (32–36). WGS is now positioned to become an essential primary platform for the management of antimicrobial resistance, including detection and surveillance of emerging drug-resistant microorganisms (33). While WGS is still a novel tool for the growing challenge of infectious diseases, broad applications of this technology beyond bacterial and viral infections have been swiftly embraced, and WGS has recently been used to study separate outbreaks of human fungal infections using whole-genome SNP phylogenetic analysis (37–39). Therefore, WGS is now poised to overtake other typing methods due to the high resolution of the data generated and the relatively low labor and monetary costs.

In this study, we determined the genetic background upon which *cyp51A* mutations occur by sequencing a diverse panel of clinical and environmental isolates of this fungus, and by doing so, we have demonstrated the utility of WGS to describe the evolutionary processes that underpin the emergence of azole resistance in *A. fumigatus*.

## RESULTS

### Species identification.

Twenty-two isolates were evaluated in our study (Table 1). The isolates were identified as *A. fumigatus* sensu stricto based on either sequencing of the internal transcribed spacer (ITS) region and amplification of parts of the β-tubulin and calmodulin genes (for the eight isolates from India and eight isolates from the Netherlands) or matrix-assisted laser desorption ionization–time of flight mass spectrometry (MALDI-TOF MS) (for the six isolates obtained from Leeds, United Kingdom). All twenty-two isolates and two control strains (NCPF 7097/AF65 and NCPF 7367/AF293) were further analyzed using whole-genome sequencing (WGS).

**TABLE 1:** Clinical and environmental isolates of *A. fumigatus* used in this study and details of alignments

Country, city	Source	Yr of isolation	Collector^a^	Culture reference	Triazole resistance^b^	No. of reads aligned (millions)	Depth of coverage (×)	% of reference genome covered^c^
UK, Leeds	Clinical	2012	R.C.B.	12-7505446	Resistant	51.9	174.1	94.4
UK, Leeds	Clinical	2012	R.C.B.	12-7505220	Resistant	48.3	161.9	95.0
UK, Leeds	Clinical	2009	R.C.B.	09-7500806	Susceptible	43.6	146.7	95.2
UK, Leeds	Clinical	2012	R.C.B.	12-7504652	Susceptible	43.7	145.6	93.1
UK, Leeds	Clinical	2012	R.C.B.	12-7504462	Susceptible	47.4	158.8	95.4
UK, Leeds	Clinical	2012	R.C.B.	12-7505054	Susceptible	50.1	168.7	93.2
Netherlands, Nijmegen	Clinical	2003	J.F.M.	08-12-12-13	Resistant	34.9	117.5	93.2
Netherlands, Nijmegen	Clinical	2005	J.F.M.	08-36-03-25	Resistant	45.2	152.5	94.8
Netherlands, Nijmegen	Clinical	2004	J.F.M.	08-31-08-91	Resistant	45.8	153.6	94.0
Netherlands, Berghem	Environmental	2008	J.F.M.	08-19-02-61	Resistant	51.7	173.2	93.7
Netherlands, Berghem	Environmental	2008	J.F.M.	08-19-02-30	Susceptible	44.5	150.1	94.8
Netherlands, Nijmegen	Clinical	2010	J.F.M.	10-01-02-27	Resistant	46.5	155.9	94.5
Netherlands, Nijmegen	Environmental	2008	J.F.M.	08-19-02-46	Resistant	48.6	163.1	93.8
Netherlands, Nijmegen	Environmental	2008	J.F.M.	08-19-02-10	Resistant	51.8	173.6	93.5
India, Delhi	Clinical	2009	A.C.	Afu 942/09	Resistant	44.2	148.1	93.6
India, Delhi	Clinical	2009	A.C.	Afu 1042/09	Resistant	41.8	139.8	93.5
India, Delhi	Clinical	2011	A.C.	Afu 343/P/11	Resistant	38.4	128.9	94.8
India, Delhi	Clinical	2012	A.C.	Afu 591/12	Resistant	34.4	115.4	93.5
India, Delhi	Environmental	2011	A.C.	Afu 124/E11	Resistant	50	167.1	93.6
India, Bihar	Environmental	2011	A.C.	Afu 166/E11	Resistant	42.8	143.6	93.6
India, Bihar	Environmental	2011	A.C.	Afu 257/E11	Resistant	33.8	113.6	93.5
India, Delhi	Environmental	2011	A.C.	Afu 218/E11	Resistant	46.5	155.8	93.5
UK	Clinical	1997	NCPF	AF65 (NCPF 7097)	Susceptible	57.5	192.8	95.1
UK	Clinical	1993	NCPF	AF293 (NCPF 7367)	Susceptible	49.3	164.5	98.1

^a^ R.C.B., Richard C. Barton; J.F.M., Jacques F. Meis, A.C., Anuradha Chowdhary; NCPF, National Collection of Pathogenic Fungi.

^b^ Resistance to triazole antifungal drugs was defined as an itraconazole MIC of >2 mg/liter using CLSI broth microdilution methods.

^c^ The AF293 genome was the reference genome for the number of reads aligned, the corresponding depth of coverage, and the percentage of the reference genome covered by reads.

### Antifungal susceptibility testing.

Antifungal susceptibility testing (Table 2) showed that 18 isolates had MICs above the established epidemiological cutoff value for itraconazole (≥2 mg/liter). All of the wild-type isolates revealed low MICs for azole antifungals.

**TABLE 2:** *In vitro* antifungal susceptibility profiles of *A. fumigatus* isolates and corresponding SNPs in *cyp51A* with nonsynonymous substitutions

Culture reference	MIC (mg/liter) of^a^:								*cyp51A* amino acid substitution at position:								Resistance marker
	ITC	VOR	POS	ISA	CAS	MFG	AFG	AMB	F46	L98	M172	N248	D255	S297	E427	F495	
12-7505446	>16	1	0.5	ND	0.125	<0.015	<0.015	0.25	Y	H	V	T	E	S	K	F	TR_34_/L98H
12-7505220	>16	1	0.5	ND	0.125	<0.015	<0.015	0.5	Y	H	V	T	E	S	K	F	TR_34_/L98H
09-7500806	1	0.25	0.06	ND	0.06	<0.015	<0.015	0.5	Y	L	V	T	E	S	K	F	None
12-7504652	1	0.5	0.25	ND	0.125	<0.015	<0.015	0.25	Y	L	V	T	E	S	K	F	None
12-7504462	0.5	0.125	0.06	ND	0.125	<0.015	<0.015	0.5	Y	L	V	T	E	S	K	F	None
12-7505054	0.5	0.5	0.06	ND	0.5	0.063	0.06	0.5	Y	L	V	T	E	S	K	F	None
08-12-12-13	>16	1	1	16	0.5	0.063	0.031	0.25	Y	H	V	T	E	T	K	I	TR_34_/L98H
08-36-03-25	>16	1	0.5	8	0.5	0.008	0.008	0.5	Y	H	V	T	E	T	K	I	TR_34_/L98H
08-31-08-91	>16	4	1	8	0.25	0.031	0.016	0.5	Y	H	V	T	E	S	K	F	TR_34_/L98H
08-19-02-61	>16	2	0.25	4	0.5	0.031	0.008	0.25	Y	H	V	T	E	S	K	F	TR_34_/L98H
08-19-02-30	0.25	0.5	0.06	0.5	0.5	0.016	0.016	0.5	Y	L	V	T	E	S	K	F	None
10-01-02-27	>16	4	0.5	4	0.5	0.016	0.016	0.5	Y	H	V	T	E	S	K	F	TR_34_/L98H
08-19-02-46	>16	4	0.5	4	0.5	0.016	0.016	0.5	Y	H	V	T	E	S	K	F	TR_34_/L98H
08-19-02-10	>16	2	0.5	4	0.5	0.031	0.016	0.25	Y	H	V	T	E	S	K	F	TR_34_/L98H
Afu 942/09	>16	2	2	8	0.125	<0.015	<0.015	0.25	Y	H	V	T	E	S	K	F	TR_34_/L98H
Afu 1042/09	>16	2	2	8	0.06	<0.015	<0.015	0.25	Y	H	V	T	E	S	K	F	TR_34_/L98H
Afu 343/P/11	>16	8	>8	>8	0.125	<0.015	<0.015	0.125	Y	H	V	T	E	S	K	F	TR_34_/L98H
Afu 591/12	>16	8	>8	2	0.25	≤0.015	0.06	0.5	Y	H	V	T	E	S	K	F	TR_34_/L98H
Afu 124/E11	>16	8	1	8	0.06	<0.015	<0.015	0.125	Y	H	V	T	E	S	K	F	TR_34_/L98H
Afu 166/E11	16	16	2	8	ND	ND	ND	ND	Y	H	V	T	E	S	K	F	TR_34_/L98H
Afu 257/E11	>16	8	1	>8	0.125	<0.015	<0.015	0.125	Y	H	V	T	E	S	K	F	TR_34_/L98H
Afu 218/E11	>16	8	1	>8	0.125	<0.015	<0.015	0.125	Y	H	V	T	E	S	K	F	TR_34_/L98H
AF65	0.5	0.125	0.06	ND	0.125	<0.015	<0.015	0.5	Y	L	V	T	E	S	K	F	None
AF293	0.5	0.25	0.06	ND	0.125	<0.015	<0.015	0.25	F	L	M	N	D	S	E	F	None

^a^ ITC, itraconazole; VOR, voriconazole; POS, posaconazole; ISA, isavuconazole; CAS, caspofungin; MFG, micafungin; AFG, anidulafungin; AMB, amphotericin B; ND, not done.

### Sequence analysis.

All 24 genome sequences mapped to >92% of the *de novo* AF293 reference genome with >100× coverage for all isolates (average of 152.7×) (Table 1). The raw sequence reads covered at least 93.1% of the AF293 reference genome for all isolates (Table 1); 98.1% of the AF293 reference genome was covered by sequence reads from the resequenced AF293 isolate. We found that 73 SNPs were also called in the resequenced AF293 isolate (data not shown), reflecting mutations due to repeated subculture (40) or sequencing errors.

**FIG 1:**
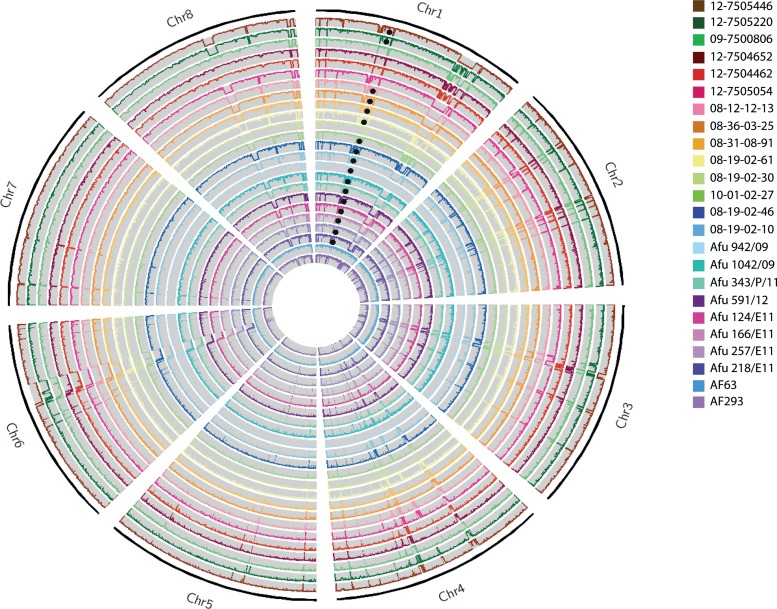
Circos (72) image of normalized whole-genome depth of coverage of all 24 *A. fumigatus* isolates (plotted as listed in the key), averaged over 10,000-bp bins. Black circles mark the presence of the TR_34_/L98H mutation. Chromosomes 1 and 6 show large deletions spanning >300 kbp in most isolates, except AF65 and AF293, while chromosome 8 displays a 60-kbp deletion in all isolates except AF65, 09-7500806, 12-7504652, and 12-7504462.

The normalized whole-genome depth of coverage was plotted to observe any possible ploidy events (Fig. 1). While none of the 24 *A. fumigatus* isolates sequenced in this study displayed copy number variation (CNV) or chromosomal CNVs, large-scale deletions in multiple chromosomes were observed. Surprisingly, deletions of over 300,000 bp were seen in all isolates except AF293 and AF65 in both chromosomes 1 and 6, accounting for the losses of 129 and 124 genes, respectively. No enrichment for a particular biological process or function was found for the genes deleted in either chromosome. However, three genes within these deletions have known functions associated with azole antifungals: Afu1g14330, an ABC transporter involved in fluconazole transport; Afu1g14050, an F-box protein whose transcript is induced by voriconazole; and Afu6g10130, an uncharacterized open reading frame (ORF) whose transcript is downregulated in response to voriconazole. A smaller deletion of 60,000 bp was observed in chromosome 8 in all isolates except 09-7500806, 12-7504652, 12-7504462, and AF65; this deletion covered a region of the genome encoding 16 genes. As for the other deletions, there was no enrichment for biological process or function in this set of deleted genes.

### SNP analysis.

Among all 24 genomes, a total of 1,895,038 SNPs were identified, 217,498 of which were common to one or more isolates. All SNPs in *cyp51A* that caused a nonsynonymous amino acid substitution are summarized in Table 2. The genomes of 16 itraconazole (ITC)-resistant isolates showed the presence of the L98H substitution. The genomes of six azole-susceptible isolates (09-7500806, 12-7504652, 12-7504462, 12-7505054, 08-19-02-30, and AF65) and one control isolate, the AF293 reference genome, had no such mutations. With the exception of L98H, the SNPs detected in *cyp51A*, including F495I, K427E, S297T, E255D, T248N, V172M, and Y46F, have previously been observed among both azole-sensitive and -resistant *A. fumigatus* strains. As shown by the data in Table 2, all 17 isolates for which the itraconazole MICs were ≥4 mg/liter had the L98H amino acid substitution in addition to the 34-base-pair nucleotide tandem repeat in their promoter region that has been previously associated with itraconazole resistance. All of the isolates for which itraconazole MICs were ≤1 mg/liter had a wild-type (i.e., that seen in reference strain AF293) *cyp51A* amino acid sequence or had the Y46F, V172M, T248N, E255D, S297T, K427E, and F495I amino acid substitutions that were present in both fully susceptible and resistant strains.

### Phylogenetic analysis.

**FIG 2:**
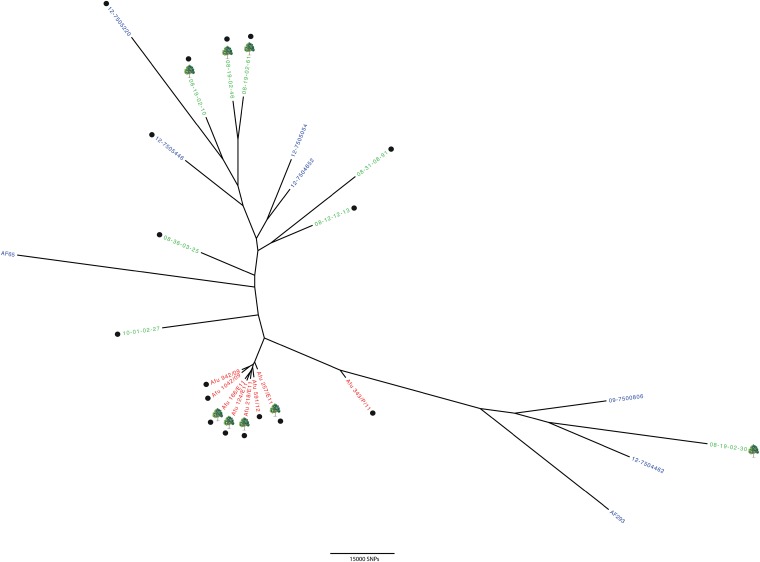
Phylogenetic analysis of *A. fumigatus* isolates representing azole-resistant and -susceptible genotypes from India, the Netherlands, and the United Kingdom. Bootstrap analysis was performed on WGST SNP data from 24 *A. fumigatus* genomes to generate an unrooted maximum-likelihood phylogeny, with all branches supported to 87% or higher. Isolates are color coded according to country of origin (red, India; green, the Netherlands; blue, United Kingdom), and environmental isolates are indicated by a tree symbol. Isolates marked with a black circle contain the TR_34_/L98H mutation in the *cyp51A* gene. Branch lengths represent the numbers of SNPs between taxa.

The 24 genomes were analyzed by whole-genome SNP phylogenetic analysis using RAxML (41). Phylogenetic analysis showed all Indian isolates to be very highly related to each other, much more so than isolates from the Netherlands or the United Kingdom (Fig. 2); this is with the exception of Afu 343/P/11, which is a clinical isolate and may have been acquired somewhere other than India (for instance through migrant working or tourism). Except for the tightly clustered isolates from India, there was no discernible relationship between the geographical location and the genotype of the cultured isolate. The phylogeny showed that clinical and environmental isolates were genotypically indistinguishable. Furthermore, phylogenetic analysis was unable to discriminate between azole-resistant and azole-susceptible strains, showing that the TR_34_/L98H allele occurs in diverse genetic backgrounds for both clinical and environmental isolates.

### Genetic diversity.

When compared against the reference isolate AF293, isolates from the Netherlands and the United Kingdom had 155,728 and 148,979 high-confidence SNPs, respectively, whereas there were 83,447 high-confidence SNPs within the Indian isolates. The average pairwise SNP diversity was higher in isolates collected from the United Kingdom (27,914) and the Netherlands (23,623) than in those from India (5,100), showing that this population has less genetic diversity despite being collected across a wider geographic range. Interestingly, 72,065 SNPs were found to be common to all three countries, accounting for 86.36% of all Indian SNPs. However, as only ~2,500 SNPs (Table 3) separate the main cluster of Indian isolates (excluding the outlier Afu 343/P/11), it is clear that the Indian isolates are genetically depauperate, manifesting less than 10% of the diversity seen in Europe.

**TABLE 3:** SNP differences between each Indian *A. fumigatus* isolate, shown as unique SNPs between isolates

Isolate	No. of SNP differences between isolate to left and:							
	Afu 942/09	Afu 1042/09	Afu 343/P/11	Afu 591/12	Afu 124/E11	Afu 166/E11	Afu 257/E11	Afu 218/E11
Afu 942/09		2207	21,150	3067	1954	2406	2928	2353
Afu 1042/09	2233		21,261	3232	2148	2625	3100	2617
Afu 343/P/11	5136	5221		5479	4861	5017	5491	4941
Afu 591/12	1943	2082	20,369		1484	1704	2401	1615
Afu 124/E11	2564	2732	21,485	3218		2383	3359	2331
Afu 166/E11	2371	2564	20,996	2793	1738		2950	1991
Afu 257/E11	1645	1791	20,222	2242	1466	1702		1623
Afu 218/E11	2539	2777	21,141	2925	1907	2212	3092	

To evaluate the genetic divergence among populations, Wright’s fixation indexes (*F*_ST_) were calculated for pairs of populations. The pairwise *F*_ST_ value for the United Kingdom and the Netherlands was zero, implying complete panmixis and interbreeding between these two populations. This finding of intermixed diversity among these two populations is supported by phylogenetic analysis (Fig. 2).

Pairwise *F*_ST_ values between Indian, United Kingdom, and Netherlands populations showed that there was a pronounced phylogeographic structure at the continental level, with an *F*_ST_ value of 0.314 for India versus the Netherlands and an *F*_ST_ value of 0.381 for India versus the United Kingdom.

### Recombination analysis.

**FIG 3:**
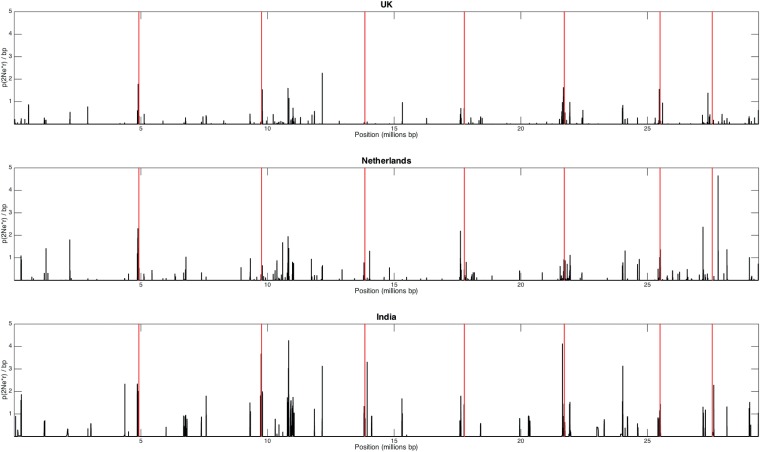
Recombination analysis, using LDhat interval (42), of *A. fumigatus* isolates from the United Kingdom, the Netherlands, and India. The black peaks represent the recombination rate across the whole *A. fumigatus* genome, and the vertical red lines mark the chromosome boundaries.

Population-wide recombination rates were estimated for each population (defined as a single country) using the program LDhat (42), which identifies patterns of linkage disequilibrium using Hudson’s composite likelihood method. The interval method used in this study specifically estimates a variable recombination rate using a Bayesian reversible-jump Markov chain Monte Carlo method under a crossing-over model. Evidence for the frequency of recombination in each population is presented in Fig. 3; the greater numbers of recombination hot spots in the United Kingdom and the Netherlands data reflect the high genetic diversity (Fig. 3). Given the lower genetic diversity observed in India (in terms of SNP numbers and also as seen in the phylogenetic analysis in Fig. 2), we anticipated this data set to be highly clonal, but on the contrary, the LDhat analysis (Fig. 3) detected similar numbers of recombination hot spots for the United Kingdom and the Netherlands, implying that the Indian data set is derived from isolates that are undergoing sexual or parasexual recombination. Overall, the rate of recombination in the Indian population (ρ = 0.0726/bp^−1^) was significantly higher (calculated using a *t* test) than the recombination rates observed in United Kingdom and Netherlands populations (ρ = 0.0070/bp^−1^ and ρ = 0.0104/bp^−1^, respectively).

In order to refute the existence of clonality in the Indian data set, we estimated the index of association (I_A_) and the modified statistic rBarD (73) to confirm the presence of recombination within each population of this data set. We assumed that the null hypothesis of no linkage disequilibrium and, therefore, no recombination would not be rejected if the resulting values of both statistics were not significantly different from the distribution of values obtained from 999 resamplings. For all three populations (India, United Kingdom, and the Netherlands), the null hypothesis could not be rejected, implying no significant linkage among loci, and we concluded, therefore, that all populations are recombining.

PCR and mining of *de novo* assembly of sequence reads has shown that both *MAT1-1* and *MAT1-2* loci are present in all populations investigated here (Table 4). While a chi-square test showed no significant difference in the frequency of either locus in the United Kingdom and Netherlands populations, a bias was observed in the Indian population, which contained significantly fewer *MAT1-2* idiomorphs (*P* < 0.0304).

**TABLE 4:** Distribution of MAT1-1 and MAT1-2 idiomorphs (mating types) among isolates of *A. fumigatus* from three countries

Country	% (no. of isolates) with mating type		Chi-square value
	MAT1-1	MAT1-2	
United Kingdom	66.6 (4)	33.4 (2)	0.41
The Netherlands	62.5 (5)	37.5 (3)	0.47
India	87.5 (7)	12.5 (1)	0.034

## DISCUSSION

To investigate the role whole-genome sequencing can play in the detection of azole resistance in *A. fumigatus*, we sequenced the genomes of 24 clinical and environmental isolates obtained from India, the Netherlands, and the United Kingdom. In the present study, we aimed to verify the applicability of using WGS to characterize known mutations in the *cyp51A* locus and to determine the genome-wide patterns of genetic diversity upon which these mutations reside.

We were able to characterize the genotype of *cyp51A* for all isolates in the study with high confidence. Of these, sixteen isolates with itraconazole MICs of ≥4 mg/liter had the L98H amino acid substitution accompanied by a characteristic 34-base-pair nucleotide tandem repeat in their promoter region. We did not detect any amino acid substitutions associated with resistance in *cyp51A* genes in the strains with itraconazole MICs of ≤1 mg/liter. Several mutations specific to the *cyp51A* gene, such as substitutions at codons G54, G138, P216, F219, M220, and G448, have also been described as responsible for resistance to azoles in clinical isolates of *A. fumigatus* (14–18), though none of these mutations were present in our analyzed sample collection. Interestingly, and in contrast with previous observations where all TR_34_/L98H mutations are accompanied by a pair of amino acid substitutions, S297T and F495I (44), only two of our 17 isolates with TR_34_/L98H showed S297T and F495I substitutions. This suggests that intragenic recombination in *cyp51A* may have occurred to unlink these mutations in this set of isolates. Whether or not this means that there have been multiple origins of the TR_34_/L98H allele will require broader WGS surveys; however, the observation that this pair of mutations has never been found unlinked to date suggests that the probability of *de novo* origination for this allele is low to nonexistent. In support of this interpretation, it is worth noting that a novel environmental *cyp51A*-mediated resistance mechanism (TR_46_/Y121F/T289A) was recently reported in the Netherlands (25), Belgium (26), Denmark (27), Germany (28), Tanzania (7), and India (29), superimposed on a more homogenous genetic background than TR_34_/L98H. This recently detected resistance mechanism has been observed to rapidly increase in frequency in Dutch hospitals and homes, where it is associated with voriconazole therapy failure (6, 25). Therefore, it appears that the contemporary rise in the frequency of TR_34_/L98H is not a unique event and that the emergence of *A. fumigatus* isolates in nature showing *cyp51A*-associated resistance alleles is an ongoing phenomenon.

Our demonstration of high levels of SNP diversity and a panmictic population structure within the United Kingdom and Netherlands is testament to the ability of this highly aerosolized (airborne) fungus to passively disperse. Our findings suggest that the TR_34_/L98H allele has existed long enough for the twin forces of recombination and gene flow to homogenize the genetic background upon which it occurs in Europe. The short branch lengths and high numbers of shared SNPs show that Indian isolates of *A. fumigatus* are broadly similar to those in Europe. However, and in sharp contrast, the Indian isolates show a far higher level of relatedness, manifesting less than 10% of the genetic diversity that is observed within the Netherlands and United Kingdom. While we have not sampled enough of the population space to delineate the full population structure and our collection is biased to isolates that are azole resistant, the *A. fumigatus* isolates from India that we sequenced were collected from locations separated by over 1,000 km. This suggests that there has been a recent expansion of this genotype within India and it is likely not a coincidence that these genotypes all contain the TR_34_/L98H allele. To further investigate the possibility that this allele has been introduced into the Indian genetic background recently, further sampling and sequencing of both azole-resistant and azole-sensitive isolates from this region are needed to clarify our finding.

There have been significant advances recently in our understanding of azole resistance mechanisms in other pathogenic fungi, such as *Candida albicans* (45) and *Cryptococcus neoformans* (46). In both species, aneuploidy for the chromosome bearing the *ERG11* gene has been reported and found to be integral to antifungal resistance. We did not detect any chromosomal aneuploidy or copy number variation for *cyp51A* or *cyp51B*, the ortholog of *ERG11*, in any of the *A. fumigatus* isolates investigated here. However, we did detect large deletions in chromosomes 1, 6, and 8 in many of the isolates (Fig. 1). Analyzing the gene content of the deleted regions revealed two genes in chromosome 1 (Afu1g14330, an ABC transporter, and Afu1g14050, encoding an F-box protein) and one gene in chromosome 6 (Afu6g10130, uncharacterized but downregulated in response to voriconazole) that had a known function associated with azole antifungals. However, given the large-scale nature of the deletions (both over 300,000 bp in size), we do not believe that these deletions are part of an evolutionary process that is enabling *A. fumigatus* to evolve resistance to azole antifungals. Indeed, we see no deletions specific to either azole-sensitive or azole-resistant isolates, suggesting that these deletions occurred in response to a different process. Deletions on this scale have not previously been reported in *A. fumigatus*, representing an opportunity for further investigation.

While most mutations in azole-resistant *A. fumigatus* isolates were single nucleotide substitutions in the target gene (*cyp51A*), mutations at other genes have been reported as conferring resistance to azoles in this fungus. Furthermore, other resistance mechanisms could be responsible for azole resistance in *A. fumigatus*. The non-*cyp51A*-mediated resistance can potentially be explained by alternative resistance mechanisms that have been described previously, including (i) higher basal expression of the *cdr1B* efflux transporter (47), (ii) a mutation in the CCAAT-binding transcription factor complex subunit *HapE* (48), which was not seen in our data, and (iii) high *cyp51B* expression (49). A recent publication reported drug resistance due to RNA interference (RNAi)-dependent epimutations in *Mucor circinelloides* (50); we found five orthologs of genes involved in the RNAi pathway in *Aspergillus nidulans*, *Cryptococcus neoformans*, and *Schizosaccharomyces pombe* present in *A. fumigatus* isolate 09-7500806. These five genes (Afu3g11010, Afu8g05280, Afu5g11790, Afu4g02930, and Afu5g11440) all had protein domains associated with RNA silencing: PIWI and PAZ domains were present in both Afu3g11010 and Afu8g05280, Dicer domains were present in both Afu5g11790 and Afu4g02930, and a domain for Argonaute small interfering RNA (siRNA) chaperone complex subunit Arb1 was present in Afu5g11440. Transcriptome profiling has revealed that all five genes are expressed in *A. fumigatus* A1163 (51). While these five genes do not represent the full RNAi pathway, we believe that epigenetic regulation could be involved in mediating antifungal drug resistance within *A. fumigatus.*

The limited set of mutations in *cyp51A* conferring resistance makes them relatively simple targets for molecular detection because their roles in resistance are known *a priori*. However, the evolution of novel azole resistance mechanisms, such as has been seen for TR_46_/Y121F/T289A and *cdr1B*, makes the detection of these “known unknowns” much more challenging. As our method captures the entirety of SNP diversity for each isolate of *A. fumigatus*, it is theoretically possible to determine the contribution of each nucleotide in the genome to the generation of the resistance phenotype within the framework of a genome-wide association study (GWAS). This “hypothesis-free” approach will ultimately extend the WGS-enabled description of known mutations, such as those reported here, to describe the full suite of drug resistance alleles and their epistatic interactions that occur in *A. fumigatus*. However, before this ambition is realized, significant hurdles need to be addressed. Most fundamentally, a description of the genome-wide global population structure of *A. fumigatus* needs to be undertaken in order to delineate the essential population parameters that underpin a GWAS; namely, the extent of population genetic structure and genome-wide rates of linkage disequilibrium. From the limited set of isolates that we sequenced, it is already evident that genome-wide recombination occurs and that SNPs are shared across the United Kingdom, the Netherlands, and India, as well as within these countries. This is an encouraging finding, especially as mixed-model GWAS frameworks are now being used and shown to perform well when handling structured populations by estimating the phenotypic covariance due to genetic relatedness (43). Although GWAS has not yet been broadly applied to microbial populations, a recent study by Sheppard et al. (53) illustrated the requirement for novel techniques in order to take these population genetic variables into account, and similar approaches are likely amenable to analyzing fungal genome-wide SNP datasets such as we have described here.

We have shown the population of *A. fumigatus* to be broadly panmictic and recombinogenic, a phenomenon that has been previously observed and confirmed by independent groups (54–57). The process of recombination is expected to accelerate adaptation to novel environmental conditions, as has previously been described in the related nonpathogenic fungus *Aspergillus nidulans* (52). Furthermore, while there were fewer recombination hot spots observed within the Indian population, the genome-wide population recombination rate was higher than that seen in the United Kingdom and the Netherlands. Seven of eight Indian isolates were typed as being *MAT1-1*, with the chi-square test showing that this bias is statistically significant. Together, these findings provide tentative evidence that an outcrossing event may have resulted in the origin of the Indian genotypic cluster, and it is worth speculating that the resulting *MAT1-1* TR_34_/L98 progeny are undergoing a rapid selective sweep by virtue of being highly fit. Clustered sexually recombining neighborhoods have previously been described in a related pathogenic fungus, *Talaromyces* (*Penicillium*) *marneffei* (58), suggesting that this type of fungal population structure may be more common than previously recognized. Whether our findings reflect the process of natural selection in *A. fumigatus* populations in response to the widespread use of agricultural azoles in Europe and India remains to be seen; however, the genomic approaches that we have detailed here will clearly underpin future research to decipher the population structure of *A. fumigatus* and map the future evolutionary trajectory of clinically relevant phenotypes.

## MATERIALS AND METHODS

### Isolates.

Twenty-four *A. fumigatus* isolates representing three geographical locations were included in the analysis (Table 1). Six clinical isolates, 12-7505446, 12-7505220, 09-7500806, 12-7504652, 12-7504462, and 12-7505054, were obtained from the Mycology Reference Centre, Leeds Teaching Hospitals National Health Service Trust, Leeds, United Kingdom. The eight isolates from the Netherlands included four clinical isolates (08-12-12-13, 08-36-03-25, 08-31-08-91, and 10-01-02-27) and four environmental soil isolates (08-19-02-61, 08-19-02-30, 08-19-02-46, and 08-19-02-10). Of the eight Indian isolates included in this study, four were clinical (Afu 942/09, Afu 1042/09, Afu 343/P/11, and Afu 591/12) and the remaining four (Afu 124/E11, Afu 166/E11, Afu 257/E11, and Afu 218/E11) originated from environmental soil sources. Additionally, the following two control isolates were included for comparison. (i) AF293 (NCPF 7367; National Collection of Pathogenic Fungi at Mycology Reference Laboratory, Public Health England, Bristol, United Kingdom) is a clinical isolate initially cultured in 1993 from lung tissue of a neutropenic patient with invasive aspergillosis in the United Kingdom; the whole-genome sequence of this strain was published in 2005 by Nierman et al. (59). (ii) AF65 is a clinical isolate originally cultured from a lung biopsy specimen from a patient with acute leukemia; this isolate was deposited at the NCPF in 1994 under accession number NCPF 7097 (60).

### *Aspergillus fumigatus* identification

All 24 isolates were identified by macroscopic and microscopic morphological characteristics as *A. fumigatus* species complex; growth at 50°C differentiated *A. fumigatus* from *Aspergillus lentulus*. Six strains from Leeds, United Kingdom, were further identified by matrix-assisted laser desorption ionization–time of flight mass spectrometry (MALDI-TOF MS), using a Bruker Daltonics BioTyper (Bruker, Bremen, Germany). All of the *A. fumigatus* isolates obtained from the Netherlands (*n* = 8) and India (*n* = 8) were previously described as *A. fumigatus* by sequencing of the internal transcribed spacer (ITS) region. In addition, the presence of any cryptic species within *Aspergillus* section *Fumigati* among these isolates was previously excluded by amplification of parts of the β-tubulin and calmodulin genes (61).

### Antifungal susceptibility testing.

The *in vitro* antifungal susceptibility profiles of triazole antifungal drugs, determined according to the CLSI M38-A2 broth microdilution method (62), were previously described and published for isolates from India and the Netherlands (61). MICs were interpreted based on proposed breakpoints (63). For six isolates, the MICs of itraconazole were primarily determined at Mycology Reference Centre, Leeds, United Kingdom, using E test strips (bioMérieux, Marcy l’Etoile, France) on RPMI 1640 agar supplemented with 2% glucose, according to the manufacturer’s instructions, and the results were interpreted as described by Pfaller et al. (64). To ensure consistency of results, MICs for all 24 isolates were further confirmed using the CLSI M38-A2 broth microdilution method.

### Conidial harvest.

All fungal isolates were subcultured on potato dextrose agar (PDA) plates and incubated at 35°C for 5 days until sporulation. Stock conidial suspensions were prepared by washing the surface of the PDA plates with 10 ml of sterile water containing 0.05% Tween 20. The conidial suspensions were filtered using Miracloth (EMD Chemicals, San Diego, CA, United States) to remove fungal hyphae, transferred to 50-ml sterile conical tubes, and centrifuged at maximum speed (10,000 × *g*) for 10 min. The supernatants were discarded, and the pellets were resuspended in 5 ml of sterile distilled water. The concentrations of the suspended conidial stocks were determined by counting the conidia using a hemocytometer chamber at ×400 magnification. Harvested conidia at concentrations of 2 × 10^8^/ml were subjected to DNA extraction.

### DNA extraction and quality assessment.

High-molecular-weight DNA was extracted with an optimized MasterPure yeast DNA purification kit (Epicentre Biotechnologies, Cambridge, United Kingdom) with an additional bead-beating step included. Harvested conidia were homogenized using 1.0-mm-diameter zirconia/silica beads (BioSpec Products, Bartlesville, OK) in a FastPrep-24 system (MP Biomedicals, Solon, OH) at 4.5 m/s for 45 s. Genomic DNA (gDNA) was quantified using a Qubit 2.0 fluorometer and dsDNA BR (double-stranded DNA, broad-range) assay kit (Life Technologies, Carlsbad, CA). Quality control of extracted gDNA samples prior to library preparation was performed using the TapeStation 2200 system (Agilent, Santa Clara, CA) and gDNA ScreenTape assays. Purified gDNAs were stored at −20°C until further use.

### Mutation screening.

Isolates were screened for the presence of a tandem repeat insertion in the promoter region of the *cyp51A* gene, as well as for the presence of the common mutations (e.g., L98H, G54, and M220), by using a mixed-format real-time PCR as previously described (10).

### Library preparation.

Genomic DNA libraries were constructed according to protocols provided by Illumina (*TruSeq Nano DNA Sample Preparation Guide*) with the TruSeq Nano kit (Illumina, San Diego, CA). Briefly, gDNA was sheared into 350-base-pair fragments using an S220 ultrasonicator (Covaris, Woburn, MA) and AFA fiber Snap-Cap microTubes, followed by end repair and solid-phase reversible immobilization (SPRI) bead-based size selection. The final libraries were quality controlled on the TapeStation 2200 system (Agilent) with D1K ScreenTape assays (Agilent) and quantified with quantitative PCR (qPCR) on an Applied Biosystems 7300 instrument (Life Technologies) using the Kapa library quantification kit (Kapa Biosciences, Boston, MA). DNA libraries were normalized to 10 nM, and randomized indexed samples were pooled into three libraries of 8 samples.

### Illumina whole-genome sequencing.

Prepared whole-genome libraries (*n* = 24) were sequenced on three lanes of the same flow cell using a HiSeq 2500 sequencer (Illumina) at Medical Research Council Clinical Genomics Centre, Imperial College London, Hammersmith, United Kingdom, generating 100-bp paired-end reads in high-output mode. All raw reads and relevant information in this study have been submitted to the European Nucleotide Archive under project accession number PRJEB8623.

### Alignment.

Raw Illumina WGS reads were quality checked using FastQC (version 0.10.1; Babraham Institute) and aligned against the AF293 reference genome (59) using the short-read alignment component (aln) of the Burrows-Wheeler Aligner (BWA) alignment tool (65) with a quality threshold of 15. Duplicate reads were marked using Picard (version 1.112).

### Identification of SNPs.

Single-nucleotide polymorphism (SNP) detection was conducted using UnifiedGenotyper from the Genome Analysis Toolkit (GATK) package (66, 67). To ensure high confidence in the SNPs called, variants were filtered according to whether they were present in 80% of reads, the mapping quality, and the depth of coverage at each base to provide a list of high-confidence variants, as described in Rhodes et al. (31). SNPs in *cyp51A* were mapped using VCF-annotator (Broad Institute, Cambridge, MA).

### Identification of tandem repeat sequences.

Whole-genome sequence reads for each isolate were used as the input for *de novo* assembly using SPAdes 3.1.1 (68). The resulting fasta file was mined for the tandem repeat sequences.

### Phylogenetic analysis.

Whole-genome SNP data were converted into relaxed interleaved Phylip format. Maximum-likelihood trees were constructed using RAxML (41) and visualized in FigTree version 1.4.0 (http://tree.bio.ed.ac.uk/software/figtree/). The rapid bootstrap algorithm was used to bootstrap all phylogenies using 100 replicates.

### Analysis of gene diversity.

Three groups, comprising 6 isolates from the United Kingdom, 8 isolates from the Netherlands, and 8 isolates from India, were formed for the purpose of analyzing genetic diversity. The fixation statistic *F*_ST_ was calculated between each population pair using the package BEDASSLE (69) for R (70).

### Recombination analysis.

Two statistics commonly used for describing linkage disequilibrium, the index of association (I_A_) and rBarD, were estimated using Poppr 1.1.2 (71) for the statistical software R (70), using 999 resamplings of data under the null hypothesis of recombination. Only variant sites unique to each population (defined by country) were considered.

The interval program of the LDhat version 2.2 package (42) was also used to estimate population-scale recombination rates using SNP data for all 8 chromosomes of *A. fumigatus*, partitioned by their country of original isolation. The program was executed for 2 million iterations with sampling every 200 iterations after a 20,000-iteration burn-in period (as recommended in the user manual [http://ldhat.sourceforge.net/manual.pdf]). The output was summarized using LDhat stat.
