# Erratum for Abdolrasouli et al., Genomic Context of Azole Resistance Mutations in *Aspergillus fumigatus* Determined Using Whole-Genome Sequencing

**DOI:** 10.1128/mBio.00939-15

**Published:** 2015-07-14

**Authors:** Alireza Abdolrasouli, Johanna Rhodes, Mathew A. Beale, Ferry Hagen, Thomas R. Rogers, Anuradha Chowdhary, Jacques F. Meis, Darius Armstrong-James, Matthew C. Fisher

**Affiliations:** ^a^ National Heart & Lung Institute, Imperial College London, London, United Kingdom; ^b^ Department of Medical Microbiology, Charing Cross Hospital, Imperial College London, London, United Kingdom; ^c^ Department of Infectious Disease Epidemiology, Imperial College London, London, United Kingdom; ^d^ Institute of Infection and Immunity, St. George’s University of London, London, United Kingdom; ^e^ Department of Medical Microbiology and Infectious Diseases, Canisius Wilhelmina Hospital, Nijmegen, The Netherlands; ^f^ Department of Clinical Microbiology, Trinity College Dublin, Dublin, Ireland; ^g^ St. James’ Hospital, Dublin, Ireland; ^h^ Department of Medical Mycology, Vallabhbhai Patel Chest Institute, University of Delhi, Delhi, India; ^i^ Department of Medical Microbiology, Radboud University Medical Center, Nijmegen, The Netherlands

## ERRATUM

Volume 6, issue 3, doi: 10.1128/mBio.00536-15. Figure 1 incorrectly displayed the location of the TR_34_/L98H mutation in gene *cyp51a*. The revised Fig. 1 (below) shows the correct location.

**FIG 1:**
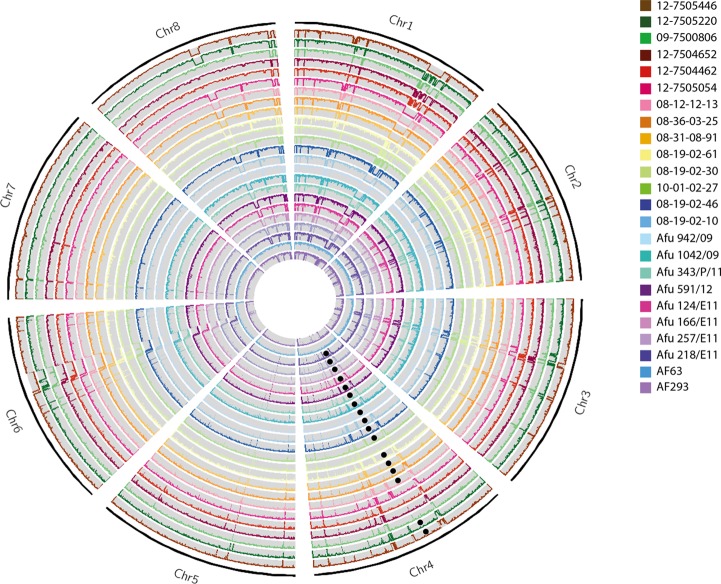
Circos (72) image of normalized whole-genome depth of coverage of all 24 *A. fumigatus* isolates (plotted as listed in the key), averaged over 10,000-bp bins. Black circles mark the presence of the TR_34_/L98H mutation. Chromosomes 1 and 6 show large deletions spanning >300 kbp in most isolates, except AF65 and AF293, while chromosome 8 displays a 60-kbp deletion in all isolates except AF65, 09-7500806, 12-7504652, and 12-7504462.

